# Promoting the Sustainability of Suicide Prevention Projects in Switzerland

**DOI:** 10.3389/phrs.2025.1607824

**Published:** 2025-04-01

**Authors:** Sophia Werdin, Eva Hollenstein, Kaspar Wyss

**Affiliations:** ^1^ Swiss Centre for International Health, Swiss Tropical and Public Health Institute, Allschwil, Switzerland; ^2^ University of Basel, Basel, Switzerland

**Keywords:** Switzerland, suicide prevention, program sustainability, intersectoral collaboration, stakeholder participation

## Abstract

**Background:**

Suicide prevention requires evidence-based measures at all prevention levels, embedded in coordinated and sustainable efforts. This policy brief aims to promote the long-term integration of project-based suicide prevention initiatives into the health system. We identify key facilitators and barriers to project sustainability and provide actionable recommendations for advancement.

**Analysis:**

This policy brief integrates evidence from a narrative review, a qualitative study with 36 suicide prevention experts, and ongoing evaluation research on suicide prevention projects to assess sustainability determinants and associated challenges. Key barriers to sustainability include evaluation gaps, insufficient collaboration, limited stakeholder awareness, organizational challenges, and financial constraints.

**Policy Options:**

To promote the sustainability of suicide prevention projects in Switzerland, we recommend: 1. Establishing a national suicide prevention website with resources and good practices. 2. Creating a guideline for multi-level collaboration and stakeholder engagement. 3. Involving target groups in project design, implementation, and evaluation. 4. Developing a sustainable financing plan early on.

**Conclusion:**

Implementing these recommendations can enhance access to structured information, foster multi-level collaboration, raise awareness, and optimize resource allocation, thereby strengthening suicide prevention in Switzerland.

## Background

Suicidal behavior is a global health issue that significantly contributes to the overall burden of disease, particularly in terms of years of life lost [1]. In 2022, Switzerland reported a suicide rate of 11.0 per 100,000 population [2]. Additionally, many more individuals attempt suicide or experience suicidal thoughts [3]. Effective suicide prevention (SP) requires timely, evidence-based, and coordinated measures that operate at multiple levels and address different target groups [4]. Interventions targeting individuals at suicide risk can be classified as secondary or tertiary prevention and are often implemented in psychiatric hospital settings. Suicide prevention projects (SPPs) are typically regional, temporary initiatives and often comprise multiple components that may have different target groups (e.g., at-risk individuals, relatives, health professionals) and may require different funding sources.

In 2016, the Swiss Federal Office of Public Health launched a national action plan aimed at reducing suicidal acts and strengthening SP throughout Switzerland [5]. While an interim report from 2021 rated the achievement of some objectives as good, it also highlighted significant room for improvement in areas such as the provision of scientific evidence and data, as well as early intervention [6]. Since 2021, the foundation Health Promotion Switzerland has co-funded four SPPs for a four-year period as part of the project support *Prevention in Healthcare.* Three projects build on the established *Attempted Suicide Short Intervention Program*, focusing on specific target groups and settings. The fourth project combines evidence-based components, such as the use of a personal safety plan, with a new self-management app for suicidal individuals. These SPPs aim to support individuals at suicide risk after hospital discharge and facilitate their transition to outpatient care.

Once scientific evidence confirms that project components effectively address the needs of their target group and potentially outperform existing approaches, their continuation should be imperative [7]. Health Promotion Switzerland defines key criteria for assessing the quality of health promotion and prevention projects [8] and has developed a concept to guide their evaluation [9]. Over the past 4 years, the Swiss Tropical and Public Health Institute and the Haute École de Travail Social Fribourg have conducted several scientific studies on the four SPPs. The findings from these external evaluations are summarized in reports that play a key role in decisions on the continuation of project components. Since the initial funding period for the four Swiss SPPs concluded in 2024, ensuring the sustainability of their components is currently of high relevance.

**FIGURE 1:**
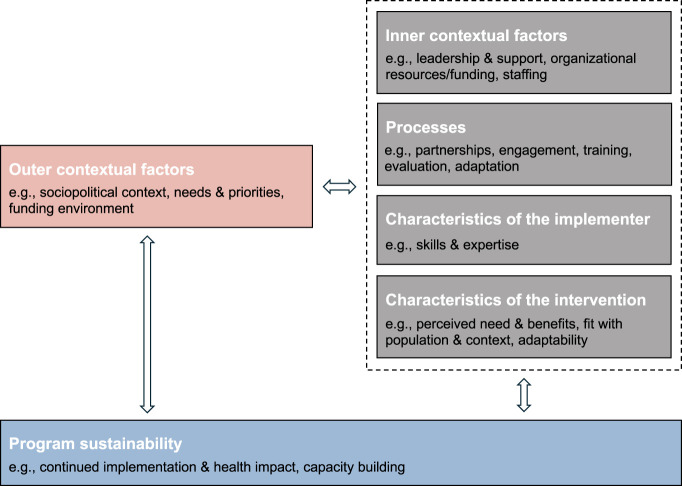
Key components and example factors of the Integrated Sustainability Framework supporting the sustainability of public health projects across community, healthcare, and social service settings (adapted and simplified from [11]) (United States, 2018).

Sustainability in this context is defined as “the continued use of program components and activities for the continued achievement of desirable program and population outcomes” [10]. The Integrated Sustainability Framework (ISF) [11] identifies key factors that support the sustainability of public health projects, categorizing them into outer and inner contextual factors, processes, characteristics of the implementer and the intervention, as well as direct sustainability factors (Figure 1). Typical determinants include community (or patient) need for the project measures, ongoing evaluation and evidence of positive outcomes, institutional support, stakeholder awareness, and the availability of sufficient financial and human resources [11, 12]. Previous research has emphasized that measures to promote sustainability should be taken from the project outset [7]. If sustainability aspects are considered too late, planning for the time after the project phase can become a major challenge.

Building on the ISF, a literature review, as well as qualitative and quantitative research in the field, this policy brief identifies key facilitators and barriers to the sustainability of SPPs. Based on these insights, we propose actionable recommendations, including implementation considerations, to promote the sustainable integration of SPP components in Switzerland.

## Analysis

This policy brief is based on diverse evidence and original research. We conducted a narrative review of international literature to identify established sustainability determinants for public health projects and their associated challenges. This review included peer-reviewed studies indexed in PubMed and grey literature, such as relevant Swiss policy documents and publications by Health Promotion Switzerland. Furthermore, findings from a qualitative study were incorporated, examining the SP landscape and the implementation of related measures in Germany, Austria, and Switzerland [13, 14]. As part of this study, 36 interviews were conducted with SP experts from policy, science, and practice, including a focus on evaluating suicide prevention measures [14] - a critical factor in ensuring their sustainability. Additionally, insights from ongoing evaluation research on the four Swiss SPPs introduced in the background section informed the development of the policy brief. For example, one study assessed the utilization and perceived usefulness of SPP components among individuals at increased suicide risk using a questionnaire [15].

Hereafter, we outline interrelated aspects that contribute to the sustainability of SPPs and describe their associated challenges.

### Evaluation and Communication of Findings

Robust evaluation is crucial for sustaining effective SPPs. However, few initiatives have been studied using rigorous methodologies, adequate sample sizes, and appropriate outcome criteria [14, 16]. Evaluating SP initiatives is challenging due to methodological, ethical, and practical barriers, such as difficulties with randomization, identifying control groups, and low response rates among individuals with (a history of) suicidal thoughts [14, 17]. Given the complex multicausality of suicidal behavior, reliably establishing a causal link between suicide rates and specific SPPs is nearly impossible [18].

Moreover, short project funding periods usually prevent long-term impact assessments, while practical factors such as feasibility, acceptability, and scalability are frequently overlooked [19]. The complexity of multi-component SPPs further complicates evaluation, and high research costs remain a major barrier to assessing effectiveness [20].

Effective communication of findings beyond academic publications and conferences is essential for integrating results into healthcare and policy [21]. Since scientific evidence is underutilized in health policymaking [22], directly conveying insights to decision-makers through mediums such as policy briefs proves to be valuable.

### Multi-Level Collaboration and Knowledge Exchange

Cross-sectoral, multidisciplinary, and interprofessional collaboration, supported by strong networks and knowledge sharing, enhances the effectiveness and sustainability of SP initiatives [23]. While specific regions like Zurich and associations such as the Initiative for Suicide Prevention in Switzerland have established good practice networks, broader collaboration across the Swiss SP landscape remains limited.

Weak connectivity among SPP initiators, both nationally and internationally, restricts access to shared resources and experiences [20]. Communication barriers, including language differences and divergent stakeholder interests, further impede collaboration. These challenges can lead to duplicated initiatives, parallel structures, and inefficient resource use, limiting the broader adoption of effective practices.

### Supportive Environment and Stakeholder Engagement

A supportive social, political, and legal environment is crucial for sustaining SP initiatives. In Switzerland, decision-making in SP, like in other public health areas, largely resides at the cantonal level with limited federal involvement. As a result, SP engagement depends on cantonal decision-makers, and regional disparities or an unsupportive political environment can undermine the sustainability of SPPs.

Engaging stakeholders across sectors such as police, media, education, and social services can significantly strengthen the sustainability of SP initiatives [24]. However, stigma, low awareness, conflicting interests, and resource constraints often hinder long-term engagement.

### Integration Into the Health System and Up-Scaling

Integrating SPP components into routine health service delivery requires alignment with (clinical) practices, payment models, and care coordination mechanisms [25]. Primary care providers face challenges such as time constraints and competing demands, complicating the integration of SPP components into routine care. In the outpatient sector, the current TARMED fee-for-service model does not adequately remunerate lengthy preventive measures. Additionally, specialized mental healthcare settings may struggle with coordinating care across providers.

Overcoming organizational barriers is key to sustainable integration, requiring anchoring across healthcare sectors in line with the integrated care approach [26]. Generalizable implementation strategies should be developed and adapted to both primary and specialized mental healthcare settings based on site-specific needs [27].

Scaling up effective SPPs beyond their initial coverage is essential for broader reach. Such an expansion must account for Switzerland’s linguistic, cultural, and cantonal health system diversity.

### Funding

Stable and adequate funding is crucial for sustaining and scaling-up SPPs [28], supporting innovation, research, and adaptability. In Switzerland, cantons are the primary funder of population-based prevention, while health insurers reimburse medical services covered under the basic benefits package, provided they meet effectiveness, appropriateness, and cost-effectiveness criteria [29]. Additional funding sources include federal grants, private foundations, non-governmental organizations, and public donations.

Securing stable funding is challenging, particularly when competing with other mental health priorities [30], dealing with fragmented funding streams for different project components, and contending with limited evaluation data.

## Policy Options

The identified challenges directly informed the development of actionable recommendations to promote the sustainability of SPPs in Switzerland. Practical considerations, including potential barriers and facilitators influencing their implementation, are detailed in Table 1. In July and August 2024, the recommendations were reviewed by thirteen different stakeholders, including SPP initiators and representatives from relevant health organizations in Switzerland, through an online survey. This stakeholder feedback was used to validate the recommendations, ensuring they are informed, relevant, practical, implementable, and tailored to meet Switzerland’s specific challenges and opportunities. The stakeholder feedback is summarized in Table 2. While the recommendations were reviewed by professional experts, the lack of input from individuals with lived experience remains a limitation of the validation process.

**TABLE 1:** Potential barriers and facilitators in implementing the proposed recommendations to promote the sustainability of suicide prevention projects in Switzerland (Switzerland, 2024).

Recommendations	Potential barriers	Potential facilitators
Recommendation 1: Establish a national suicide prevention website that features resources and information on good practice projects	Difficulty in obtaining an overview of all existing projects Limited interest of the initiators of suicide prevention projects in collaborating with other projects Challenges in designing a website that is useful for a diverse audience Human resource constraints (especially for keeping the website up to date) Resistance to change/consolidation in a new platform, fragmentation of efforts Sustaining engagement over time	Platform design and content guided by existing websites Centralization of resources Funding and policy support Cross-sectoral partnerships (e.g., including healthcare, police, media, social services, education) Participatory process in website’s design Focusing on evidence-based projects Awareness campaigns International collaboration Continuous marketing and engagement efforts Mechanisms for ongoing evaluation and incorporating user feedback
Recommendation 2: Develop a guideline for multi-level collaboration and stakeholder engagement in suicide prevention	Policy and legislative barriers Differences in the organization of suicide prevention across cantons Linguistic diversity Limited acceptance of the guideline Limited awareness and divergent interests among stakeholders	Leveraging existing good practice networks (e.g., in Zurich) Participatory process in guideline design Interprofessional working group Government and policy support Stakeholder engagement strategies Cultural and linguistic adaptability Training and capacity building
Recommendation 3: Involve target groups in project design, implementation, and evaluation	Identification of “suitable” individuals Resistance to involvement by “suitable” individuals Coordinating the involvement of the target groups Balancing the perspectives and needs Lack of expertise Keeping individuals engaged	Multiple, accessible communication channels Partnerships with non-governmental organizations, schools, and volunteers Incentives for participation Training and support for participants Feedback mechanisms Transparency about project goals, processes, and outcomes
Recommendation 4: Develop and maintain a sustainable financing plan early on	Complexity of aligning and coordinating diverse health prevention and healthcare funding streams Identifying and mobilizing funding sources Lack of evidence supporting project’s effectiveness Lack of approaches for transitioning from project funding mechanisms to routine service delivery	Diverse funding sources Public-private partnerships Comprehensive project evaluation Demonstrating the project’s value Systematic search for funding A clear plan for transitioning from a project-based to a system-wide logic Public relations

**TABLE 2:** Stakeholder feedback (N = 13) on the proposed recommendations (Switzerland, 2024).

Recommendation 1: Establish a national suicide prevention website that features resources and information on good practice projects	Relevance	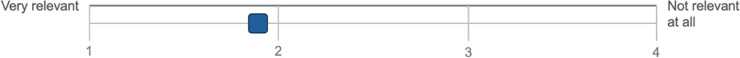
	Feasibility	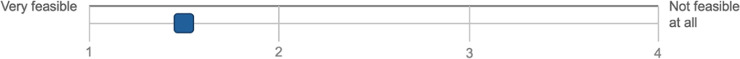
Recommendation 2: Develop a guideline for multi-level collaboration and stakeholder engagement in suicide prevention	Relevance	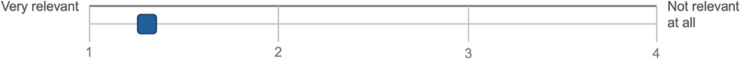
	Feasibility	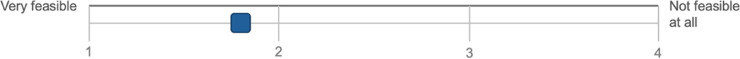
Recommendation 3: Involve target groups in project design, implementation, and evaluation	Relevance	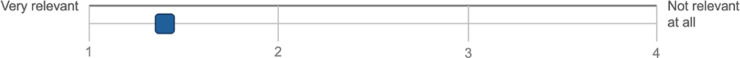
	Feasibility	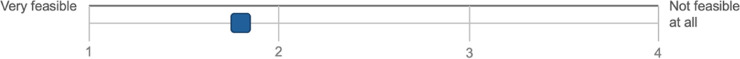
Recommendation 4: Develop and maintain a sustainable financing plan early on	Relevance	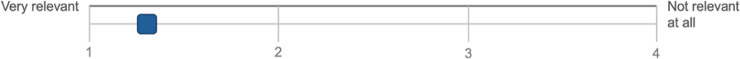
	Feasibility	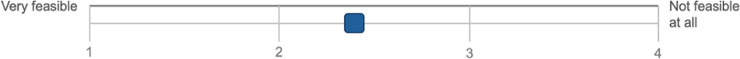
How well can these recommendations, if successfully implemented, promote the sustainability of suicide prevention projects in Switzerland?		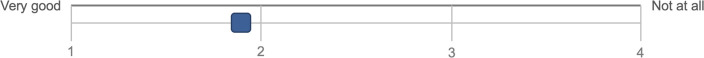
Open feedback (selection)	- *“*A shared vision, broken down into a few SMART goals, which document the multiprofessional networking and synergistic, cross-sector, and sustainable collaboration in a guideline committed by stakeholders, actors, and affected individuals/relatives, is a long but certainly worthwhile path!” - “Exactly these four aspects have contributed to the sustainability of the suicide prevention focus program in the canton of Zurich and will remain essential in the future.” - “Networking, inclusion of target groups, and long-term funding. These are key factors for me.” - “Recommendations are only implementable if the stakeholders a) accept them and b) receive sufficient financial means and personnel resources for implementation.” - “A more inclusive and integrated approach to suicide prevention is needed. This requires for sure more cooperation between professionals from various fields. While a guideline for improving cooperation can help, it is not enough. The main question is: how can we foster a shared culture of suicide prevention among professionals? Achieving this requires education and resources to enable collaboration across various contexts.”	

### Recommendation 1: Establish a National Suicide Prevention Website That Features Resources and Information on Good Practice Projects

We recommend creating a national SP website, supported by a strong promotional strategy. This platform should showcase evidence-based practices and new SPPs, providing structured information on evaluation research and findings to enhance transparency and credibility among the public and funders. In addition to Swiss initiatives, effective approaches from abroad should be included to facilitate global knowledge exchange. Good practice projects could be identified based on the quality criteria for health promotion and prevention projects established by Health Promotion Switzerland [8].

Beyond listing SPPs, the website should serve as a comprehensive SP resource hub for diverse audiences, including health professionals, individuals at suicide risk, their relatives, and the general public. It could feature evidence-based guidelines for managing individuals at suicide risk in both clinical and lay settings, list upcoming events, training opportunities, scientific updates in the field, and link support services. The website should be developed using a participatory approach, involving SP experts from policy, science, and practice, as well as individuals with lived experience and relatives. It could be managed by the Swiss Federal Office of Public Health or Health Promotion Switzerland, with support from Swiss SP societies and associations. One stakeholder suggested commissioning IPSILON, the umbrella organization for suicide prevention in Switzerland, to manage the platform via a service contract.

While there are existing websites showcasing Swiss health projects (e.g.,^1^), including SPPs, we advocate for a dedicated, centralized SP website accessible in German, French, Italian, and English. To streamline information access and avoid redundancy, existing websites should direct users to this central hub. A national SP website would ensure that individuals at suicide risk, their relatives, health professionals, and the general public can easily access structured information on available SP services and resources. Transparently showcasing good practices can help prevent the creation of parallel structures. Including contact details of project initiators would facilitate national and international knowledge sharing, networking, and collaboration. Promoting the website via multiple channels, including social media, live streams, and events, is essential to maximizing its reach and impact.

### Recommendation 2: Develop a Guideline for Multi-Level Collaboration and Stakeholder Engagement in Suicide Prevention

Effective and sustainable SPP components require strong collaboration across sectors, stakeholders, and professions, while strategic partnerships facilitate resource and knowledge sharing. An evidence-based guideline for multi-level collaboration in SP can help structure and sustain these efforts long term. To ensure lasting stakeholder engagement, the guideline should outline strategies for initiating and maintaining contact, along with methods to keep stakeholders informed and engaged. Regular multi-sectoral roundtables and annual stakeholder conferences could help maintain momentum. An interprofessional working group, including professionals from healthcare, prevention, police, and media, should be officially tasked with developing this guideline. The instrument must be adaptable to different cultural contexts and cantonal health systems in Switzerland.

Existing good practice networks, such as those under Zurich’s cantonal focus program for SP, could inform guideline development. In Germany, experts are currently developing an S3 guideline on dealing with suicidality (ID 038-028, https://register.awmf.org/de/leitlinien/detail/038-028), which also addresses interprofessional collaboration in SP. This instrument could serve as a model for creating a version tailored to Switzerland’s specific conditions while avoiding duplication. Additionally, the national SP website proposed in Recommendation 1 could serve as a hub for networking and information exchange, further strengthening stakeholder engagement and multi-level collaboration.

### Recommendation 3: Involve Target Groups in Project Design, Implementation, and Evaluation

Engaging target groups throughout a project’s lifecycle can enhance the effectiveness of SPPs. Depending on the project component, these groups may include individuals at suicide risk, their relatives, health professionals, and the general public. Effective engagement can be achieved through interviews, focus groups, surveys, or direct involvement, such as having individuals with lived experience speak at public events. This can foster a sense of ownership and commitment while ensuring SPPs are culturally adapted to community needs and characteristics.

Incorporating perspectives from individuals with lived experience and relatives during design, implementation, and evaluation can improve the relevance, practicality, and impact of SPPs. For example, training individuals with lived experience as peer supporters can strengthen outreach efforts, while sharing personal experiences and coping strategies enhances information quality, builds trust, and promotes help-seeking behavior. High acceptability, accessibility, and cultural appropriateness of SPP components increase their likelihood of being sustained and having long-term impact.

### Recommendation 4: Develop and Maintain a Sustainable Financing Plan Early on

SPPs should diversify funding sources by exploring options such as integrating suicide prevention interventions into the basic benefit package of the health insurance, leveraging cantonal health promotion programs, seeking support from foundations, and encouraging donations to enhance financial stability and longevity. Developing an early, sustainable business and financing plan supports this effort. This plan should define the project’s mission, measurable objectives, strategies, and a needs assessment to determine funding requirements. It should also outline how interventions will be sustained after the project concludes, such as integration into existing public health initiatives or hospital-based services.

Proactively seeking funding from both public and private sectors and collaborating with government agencies for public funds should begin early in the project’s lifecycle. Demonstrating relevance, acceptability, effectiveness, and scalability is key to securing long-term financing. For example, proving economic efficiency is crucial for integrating new services into the basic health insurance benefits package. Continuous process and outcome evaluation, along with transparent result-sharing, keeps stakeholders engaged and can attract new funding sources.

## Conclusion

This policy brief advocates for a multifaceted approach to promote the sustainability of SPP components in Switzerland. It underscores the importance of rigorous evaluation, strategic collaboration, participatory approaches, and sustainable funding. By outlining actionable recommendations, we provide a pragmatic path forward. Achieving long‐term impact requires coordinated action and a strengthened commitment from all stakeholders to advance suicide prevention in Switzerland.

## Footnotes

1
https://www.bag-blueprint.ch, https://www.good-practice.ch

## Abbreviations

ISF, Integrated Sustainability Framework; SP, Suicide Prevention; SPP, Suicide Prevention Project.
